# Newborn Screening for Lysosomal Storage Diseases: A Concise Review of the Literature on Screening Methods, Therapeutic Possibilities and Regional Programs

**DOI:** 10.3390/ijns3020006

**Published:** 2017-03-29

**Authors:** Peter C. J. I. Schielen, Evelien A. Kemper, Michael H. Gelb

**Affiliations:** 1 Reference Laboratory for Neonatal Screening, Centre for Infectious Diseases Research, Diagnostics and Screening, National Institute for Public Health and the Environment, 3720 BA Bilthoven, The Netherlands; 2 Department of Clinical Chemistry, IJsselland Hospital, 2906 ZC Capelle ad IJssel, The Netherlands; 3 Departments of Chemistry and Biochemistry, University of Washington, Seattle, WD 98195, USA

**Keywords:** lysosomal storage disease, tandem mass spectrometry, fluorimetry, neonatal screening, newborn screening, enzyme replacement therapy, hematopoietic stem cell transfer, pilot screening program, biomarker

## Abstract

Newborn screening for lysosomal storage diseases (LSDs) is increasingly being considered as an option. The development of analytical screening methods, of second-tier methods, and of therapeutic possibilities, are paving the way for routine screening for LSDs in the coming years. Here, we give a brief description of the current status quo, what screening methods are currently available or are in the pipeline, what is the current status of therapeutic possibilities for LSDs, what LSDs are the most obvious candidates for introduction in screening programs, and what LSDs are already part of regional or national pilot or routine screening programs worldwide.

## 1. Introduction

Lysosomal storage diseases (LSDs) are part of the group of rare inherited metabolic diseases. Treatments are available for a subset of LSDs, and it has been shown in some cases that initiation of treatment as early as possible gives the best clinical outcome. Meanwhile, there are only relatively few programs where screening for some LSDs is implemented in routine screening. Here, we review the current state of screening for LSDs, especially addressing currently available methods for analysis and therapeutic possibilities. We also give an overview of programs with some level of implementation of screening for LSDs.

## 2. What Are Lysosomal Storage Diseases

Lysosomes are intracellular organelles that breakdown and recycle a range of complex cellular components including glycosaminoglycans, sphingolipids, glycogen fragments, and proteins. The catabolic function is performed through the concerted action of approximately 60 different types of acid hydrolases. Lysosomal dysfunction leads to intra-lysosomal accumulation of the non-degraded substrates, which causes cell destruction and eventually organ damage.

LSDs are a group of more than 50 inherited metabolic disorders. Most are inherited in an autosomal recessive manner, although some are X-chromosome linked. LSDs are mostly characterized by a deficiency of specific enzymes, resulting in accumulation of specific substrates in lysosomes and producing large intracellular vacuoles (reviewed in [1,2]). LSDs are caused by mutations in genes encoding soluble acidic hydrolases, integral membrane proteins, activator proteins, transporter proteins, or non-lysosomal proteins that are necessary for lysosomal function.

LSDs are characterized by a broad spectrum of clinical phenotypes of which most are not specific for LSDs. Phenotypes depend on the type, quantity and site of storage of non-degraded material, and their diversity complicates early diagnosis. In addition to the age of onset, severity of symptoms and central nervous system manifestation can vary markedly within a single disorder. Most of the patients with LSDs are born apparently healthy, and the symptoms develop progressively. Some common symptoms include facial dysmorphism, (neuro)developmental delays, recurrent infections, muscle problems, organomegaly and skeletal changes. The diagnostic workup traditionally includes urine analyses for specific non-degraded macromolecules and enzyme activity analysis in blood cells or fibroblasts. Although LSDs are individually rare, the prevalence of the whole group of diseases is relatively high when compared to other groups of rare diseases. Combined birth frequencies of LSDs range from 7.5 per 100,000 in British Columbia to 23.5 per 100,000 live births in the United Arab Emirates, with sphingolipidoses as the most prevalent group, followed by the mucopolysaccharidoses [3].

## 3. Treatments for LSDs in Place and under Development

As LSDs comprise a broad range of diseases, affecting multiple organ systems, with uncertain etiology, both early onset and late onset, and affecting patients’ health and life-expectancy in varying ways, treatment is necessarily multidisciplinary. The clinical treatment of LSDs was recently extensively reviewed [1,4]. Historically, only palliative care was available (e.g., support therapy to manage neurological complications, ventilator support). In the last 25 years, however, the focus has been on developing therapies that correct the metabolic effects of LSDs. This may imply administering an active enzyme to replace the defective one, stabilizing an affected enzyme or correcting its proper functioning. Alternatively, the problem of accumulation of substrates is targeted, e.g., by reducing substrate synthesis or clearing of substrates from the cells. Some of these therapies are highly experimental, and some are applied in actual patient care.

### 3.1. Enzyme Replacement Therapy (ERT)

First studies demonstrating the efficacy of ERT in Gaucher disease were performed in the early 1990s. Between 2000 and 2007, ERT was also used for Fabry and Pompe disease, MPS I, II and VI, and treatment for all of these LSDs is now approved by intravenous administration of the recombinant enzyme. For five other LSDs, ERT is being tested in clinical trials both in Europe and in the U.S.A (for comprehensive reviews on ERT, see [1,4,5]. In an extensive health technology assessment on ERT licensed in the UK for Gaucher disease, Fabry disease, MPS I, II and VI and Pompe disease, it was concluded that, in general, ERT is effective to treat patients with these LSDs, although study numbers were limited [6].

Surely, these therapies have been successful to some extent, but there is significant variability in effectiveness among patients. The disease may progress after an initial phase of recovery and in tissues such as bone, cartilage and heart, there may be little effect.

One problem with ERT is its limited bio-availability. Recombinant enzymes are large molecules that do not freely diffuse across membranes and often do not reach therapeutic levels in target tissues, especially the central nervous system. Strategies to biochemically modify these recombinant enzymes to prolong their half-life, or alternatively, couple them with Trojan-horse peptides to facilitate passing the blood brain barrier, are still in a pre-clinical phase. Administering ERT intrathecally to enhance bio-availability in neural tissue has been applied in patients with MPS-I and VI [7,8].

### 3.2. Hematopoietic Stem Cell Transplantation (HSCT)

Hematopoietic stem cells, derived from donor bone marrow or umbilical cord blood may be therapeutic by repopulating tissues and secreting functional lysosomal enzymes in the extracellular space and blood. These functional enzymes cross the blood–brain barrier, and in tissues can be internalized by cells, cross-correcting the deficient enzyme activity and thus repairing the metabolic defect. HSCT is the only available therapy for Krabbe disease and has been shown to work in some other LSDs (MPS-I; [9]), but it is ineffective in several others (MPS II, III) while HSCT is no longer recommended for Gaucher [6]. Again, bone and brain are the tissues least susceptible for the beneficial effects of HSCT.

The best results are obtained when HSCT is performed early in life (see Ref. [9] for MPS-I, Ref. [10] for metachromatic leukodystrophy (MLD), and Ref. [11] for Krabbe disease). Disadvantages of HSCT include graft versus host disease, graft rejection and infection.

### 3.3. Substrate Reduction Therapy (SRT)

There is some progress with substrate reduction therapy. Miglustat is a substrate-reducing agent that is approved to treat Type 1 Gaucher and Niemann–Pick disease, and a few others are being tested in clinical trials [1,4].

### 3.4. Gene Therapy

As most LSDs are monogenic, they are potentially excellent candidates for gene therapy, especially since the technological possibilities of gene therapy are rapidly progressing. Currently, only one successful example of gene therapy is described for an LSD (for metachromatic leukodystrophy [10]).

## 4. Measuring Lysosomal Enzymatic Activities and Biomarkers in DBS

The development of high throughput assays for testing LSDs using DBS has been a challenge, but, in the past decade, considerable progress has been made. The heterogeneity of mutations in the LSDs and our poor understanding of genotype–phenotype correlations makes DNA sequencing-based and first-tier screening impractical. In addition, the lack of general and specific metabolic markers limits the use of biomarker quantification screening assays, which is mainly used in newborn screening.

The key breakthrough came when Chamoles and coworkers showed that most lysosomal enzymes are active in rehydrated DBS, thus permitting their activities to be measured with fluorimetric substrates [12–14]. Enzyme activity was measured using artificial 4-methylumbelliferyl substrates followed by quantification of the enzyme products by fluorescence. In 2006, Civallero et al. described enzymatic (fluorescence and radiometric) assays for twelve different LSD-enzymes [15]. Some years later, an optimized enzymatic assay for MPS I was published in which the problem of interference of hemoglobin was diminished [16].

At the same time, the development of electrospray ionization tandem mass spectrometry (MS/MS) for detecting LSDs was reported [17,18]. The original method involved addition of a cassette of lysosomal enzyme substrates and internal standards in buffer to a DBS punch. After incubation, the sample was processed by solid-phase extraction (passage through a small plug of silica gel) followed by infusion into the mass spectrometer. This original method was further optimized to be compatible with high throughput newborn screening laboratories [19]. The use of the internal standard, which is chemically identical to the enzymatically-generated product but differentiated in the mass spectrometer by substitution of hydrogens with deuterium, accounts for all variation in the product signal due to sample losses and mass spectrometry response. This is not possible with fluorimetric assays. The original MS/MS assays were for Pompe, Fabry, Gaucher, MPS-I, Niemann–Pick-A/B, and Krabbe diseases. More recently, MS/MS enzymatic activity assays have been developed for MPS-II, IVA, and VI [20,21] and MPS-IIIB and -VII (M.H. Gelb, unpublished). Very recently, the MS/MS method has been simplified by replacing the solid-phase extraction step with a liquid–liquid extraction step using ethyl acetate [22]. This is the method going forward including commercial production of reagents and kits.

Early pilot studies using fluorimetry or MS/MS to examine newborn screening for LSDs by measurement of enzymatic activities in DBS were reported [23–31]. These initial studies suggested that it will be feasible to screen for LSDs by measurement of lysosomal enzymatic activities in DBS.

Due to DNA sequencing of lysosomal enzyme genes not being feasible for first-tier newborn screening as mentioned above (ill-understood phenotype–genotype relations) and, in addition, costs and the current performance of fast DNA-sequencing techniques, the only other approach being considered is measurement of biomarkers for LSDs by MS/MS. These methods have been recently reviewed [32] and are discussed briefly in Section 7 below. These methods are not moving forward for first-tier NBS of LSDs either because the analysis time per sample is too long for high throughput NBS or because they give false positive rates that are not practical (3%–5% in some cases). However, these methods are expected to be extremely valuable for second-tier analysis (in screen positive cases identified by enzymatic activity assay), especially when the same DBS can be used so as to avoid patient recall and parent anxiety.

In the past 1.5 years, there has been new progress in the development of newborn screening for MLD. This LSD is caused by a deficiency in the enzyme arylsulfatase A (ARSA) that removes the sulfate from sulfatide. Direct assay of ARSA enzymatic activity in DBS is almost certainly not feasible for newborn screening of MLD because the enzyme is unstable in DBS [33] and because the pseudodeficiency problem is very severe (it seems that even 1% residual ARSA activity is sufficient to prevent symptoms from developing [34] (but see also [35] for the effect of residual activity in other LSD). The only feasible approach reported to date is the quantification of sulfatides in DBS [34]. Sulfatides accumulate to a higher extent in urine than in DBS, but urine is not normally collected in most newborn screening programs. A large-scale pilot study to evaluate the ability of sulfatide analysis in DBS by MS/MS has begun at the University of Washington (M. Gelb, unpublished). The goal is to reach ~200,000 analyzed DBS over 2–3 years.

## 5. Large Scale Pilot Studies of Newborn Screening of LSDs in DBS by Direct Enzymatic Activity Measurement Using MS/MS or Fluorimetry

The first large-scale pilot study of LSD newborn screening was carried out in Taiwan for Pompe disease [30]. The authors developed a fluorimetric assay using a standard plate reader in which three different conditions were used. The first is to measure the enzyme relevant to Pompe disease, acid α-glucosidase (GAA), at low pH in the presence of acarbose to inhibit maltase-glucoamylase that normally interferes [36]. They also measured α-glucosidase (NAG) at neutral pH and total α-glucosidase (TAG) at low pH without acarbose. These three activities were used in a ratiometric manner that was useful in partially separating Pompe-affected from pseudodeficiency newborns. Pseudodeficiencies for Pompe disease are very common in the Asian population. This was the first convincing study to show that newborn screening for Pompe disease is feasible using direct assay of GAA enzymatic activity in DBS.

The next large-scale pilot study used MS/MS to measure the activity in a single DBS punch of the enzymes relevant to Pompe, MPS-I, and Fabry diseases [28]. Results are summarized in Table 1. The studies done in the Washington and New York newborn screening laboratories use the MS/MS method, whereas the Missouri newborn screening lab uses the fluorimetric assays with a digital microfluidics platform [37]. We focus on the number of screen positives (those below the chosen cutoff) per 100,000 newborns. In all cases, a majority of the screen positives are false positives as revealed by genotyping of the relevant lysosomal enzyme gene. Among the “true” positives, the majority are asymptomatic newborns who may develop a late onset LSD. It is for this reason that we do not report false positive rates and positive predictive values (genotypes are available in the publications from the Washington newborn screening laboratory). These three large studies show the feasibility of newborn screening for several lysosomal storage diseases by direct enzymatic activity measurement in DBS. They also show that the MS/MS method gives a significantly lower number of screen positives than the fluorimetric method [21]. This is particularly clear for Pompe disease where equivalent cutoff values were used in Washington, New York and Missouri (cutoffs chosen to be just above the enzymatic activity measured in an identical set of DBS from Pompe-affected patients). Note that the number of screen positives in New York and Washington for Pompe disease are virtually identical, suggesting that there is not a large population variation across the USA. A possible reason for the improved performance of the MS/MS assay (assuming that the lower number of screen positives reflects a better positive predictive value) is given in Section 6.

Finally, considerable progress to lower the initial number of positive screening results has been achieved recently by using the CLIR database [39] together with MS/MS enzyme assays. The CLIR team reports successful use of the CLIR database to significantly reduce the false positive rates for newborn screening Krabbe, MPS-I, and Pompe disease in the state of Kentucky [40]. These are very encouraging results, and expanded use of CLIR for LSD NBS is expected in the near future.

## 6. Additional Comparison of MS/MS and Fluorimetric Methods for LSD Newborn Screening

In search of a reason for the lower rate of screen positives using MS/MS versus fluorimetry, we carefully studied the analytical range of these assays. We define the analytical range as the assay response for the quality control HIGH sample (typical of a non-affected newborn) divided by the assay response for all processes that are independent of the relevant lysosomal enzyme (i.e., various sources of background). The analytical range for MS/MS is more than an order-of-magnitude larger than for the corresponding fluorimetric assays [21,22,32]. It is reasonable to presume that the assay with the larger analytical range will give rise to a lower number of screen positives (with equivalent cutoff values) because the “scores are spread” more, thus leading to a higher assay resolution and accuracy. The analytical range of the MS/MS assay is limited by the small amount of enzyme substrate that decomposes to the enzymatic product in the heated electrospray source of the mass spectrometer. In the case of fluorimetry, the analytical range is limited by the intrinsic fluorescence of the 4-methylumbelliferyl substrate, which is a significant factor with DBS assays, in which only 1%–2% of the total substrate is converted to products [21].

A detailed comparison of the relative cost and space requirements of MS/MS and digital microfluidics fluorimeters is not included here but has been recently reported [22,32]. Space and costs are shown to be similar for both methods. Information on pricing is also available from customers who have purchased the various technologies for LSD NBS. An additional advantage of MS/MS is that the substrates are closer in structure to the natural enzyme substrates owing to the fact that incorporation of a fluorogenic group into the molecule is not required. This issue has become important for NBS of Niemann–Pick-A/B disease. Studies have shown that use of the fluorogenic substrate leads to an NBS and diagnostic pitfall because of the presence of a common mutation, Q292K, in affected patients that does not reduce the activity when the fluorogenic substrate is used but does remove the activity when the natural sphingomyelin or closely-related MS/MS substrate is used [41,42]. This is a serious issue with the fluorimetric assay since it is predicted to lead to a false-negative in ~10%–15% of the Niemann–Pick-A/B-affected newborns. Apparently, this mutation does not disrupt the binding of the artificial fluorogenic substrate to the active site of the sphingomyelinase, but it does block the binding of the natural and MS/MS substrates.

## 7. Biomarkers for LSDs

Biomarkers for LSDs have been recently reviewed [32]; we give a brief summary here. Biomarkers for LSDs tend to be metabolites that accumulate as a result of a deficient lysosomal enzyme.

Glucose tetrasaccharide has been studied as a potential biomarker for Pompe disease [43], but its use for NBS has not been suggested. This is based on the long chromatographic time needed for accurate analysis, and the report of a large number of cases where the metabolite is elevated in newborns without Pompe (personal communication with D. Millington, Duke University). Glucosylceramide, the substrate for the relevant enzyme is reported to be on average three-fold elevated in type I Gaucher patients compared to levels in normal samples [44,45]. However, even when a small number of DBS are analyzed, overlap between affected and non-affected patients is apparent. This marker appears to be valid as a second-tier approach for analysis of Gaucher disease but not for first-tier NBS. Glucosyl-sphingosine appears to be a more promising biomarker for Gaucher disease [44], but, again, it is most appropriate for second-tier analysis [45]. Psychosine (galactosyl-sphingosine) accumulates in patients with Krabbe disease, and this metabolite is turning out to be useful in the post-NBS evaluation of newborns at risk of developing Krabbe disease [46,47]. Psychosine analysis requires a lengthy chromatographic separation time, making it unsuitable for high throughput NBS. Recently, it was shown that lysosphingomyelin is elevated in DBS from Niemann–Pick-B patients [48]. A long chromatographic separation time and the fact that the elevation was only ~five-fold in affected patients suggests that this biomarker is not suitable for first-tier NBS of Niemann–Pick-A/B. Recently, a glycine-conjugated bile acid derivative was identified as a powerful biomarker for detection of Niemann–Pick-C [49]. In a limited pilot study of a few thousand DBS, the biomarker showed good specificity for the LSD arguing that an expanded pilot study is warranted. The exact biological function of the Niemann–Pick-C protein is uncertain. It is proposed to be involved in lipid transport across the lysosomal membrane, and thus an enzyme-based NBS assay is not relevant. There have been numerous reports on the MS/MS analysis of glycosaminoglycans and their fragments for evaluation of multiple types of mucopolysaccharidoses; for example, the early work of De Ruiter [50]. These methods have been recently reviewed [51,52] and are thus not discussed in detail here. A recent pilot study of glycosaminoglycan fragment analysis in ~2000 DBS was carried out in Japan [53]. The false positive rate for MPS-I and MPS-II was ~0.03%, but it rose to ~0.9% with inclusion of MPS-III. Inclusion of MPS-IVA and MPS-VI raised the false positive rate to ~3% [53]). By comparision, the false positive rate for newborn screening of MPS-I by direct assay of the relevant enzyme by MS/MS is less than 8 in 100,000 DBS (<0.008%, Table 1 and [28]). These results suggest that glycosaminoglycan analysis is most appropriate for second-tier analysis following first-tier analysis by measurement of the relevant lysosomal enzymatic activity in DBS. Glycosaminoglycan analysis can also be done on the same DBS as the enzymatic assay so as to avoid newborn recall and thus family anxiety. A second issue is that glycosaminoglycan fragment analysis requires a more elaborate pre-MS/MS sample preparation involving expensive enzymes and also an LC-MS/MS time of several minutes per sample.

## 8. Status of LSDs in Current Screening Programs

Over the years, LSDs have been candidates to be evaluated for inclusion in national screening programmes. As knowledge on the prevalence increases, screening methods and therapeutic possibilities become increasingly available, and pilot studies have been completed, LSDs are being added to national or regional screening programs.

The programme with possibly the most inclusions is the New York state screening program with about 3.3 million newborns now screened for Krabbe disease using MS/MS, leading to the identification of five infants confirmed by clinical examination to have early infantile Krabbe disease. In addition, 12–14 newborns were identified to be at high risk for developing Krabbe disease but are so far asymptomatic as far as we know [11,54]. Krabbe disease newborn screening is also in place in Missouri using fluorimetry, initially carried out by the New York newborn screening laboratory. A recent report showed that some 235,000 NBS samples were analysed identifying 40 cases with polymorphisms only (clinically not relevant), and of 54 other referrals, with eight genotypes of unknown clinical significance, and 31 with one clinically relevant mutation [36]. Missouri is also live for newborn screening of MPS-I, Gaucher, Pompe and Fabry diseases by fluorimetry. Illinois is live for newborn screening of these four LSDs plus Niemann–Pick-A/B by MS/MS. New York added live newborn screening for Pompe disease by MS/MS in October 2014. Ohio and Kentucky are live for newborn screening of MPS-I, Pompe, and Krabbe diseases by MS/MS. Other states in the USA are close to initiating live newborn screening for LSDs: Wisconsin, New Jersey, Massachussetts and others are in the planning stages (Michigan, Florida, Minnesota, Texas, Colorado).

Taiwan has an extensive program for LSD newborn screening. One of three national centers is live for Pompe, Fabry, Gaucher, MPS-I, MPS-II, MPS-IVA, and MPS-VI by MS/MS (unpublished information from The Chinese Foundation for Health, Taipei, Taiwan). As noted above, newborn screening for Pompe disease in Asia is plagued by the enormous frequency of pseudodeficiency alleles. Paul Hwu and co-workers (National Taiwan University) use a three-enzyme activity method (described above) to reduce the false positive rates; this method has now been converted from a fluorimetric to an MS/MS assay by using a series of deuterium-encoded substrates and internal standards (P. Hwu and M. Gelb, unpublished). More recently, Liao and Chiang (Chinese Foundation of Health) started to use a modified MS/MS assay for Pompe disease that leads to almost complete separation of Pompe-affected from patients carrying one or more pseudodeficiency alleles [55].

Despite the above developments, to date, Fabry and Niemann–Pick disease were deemed ‘not ready for review’ by the U.S. advisory committee on heritable disorders in newborns and children (ACHDNC). MPS I and Pompe disease, however, were recently added to the RUSP following the recommendation of the ACHDNC.

In Europe, the screening for LSDs is in its early stages. In Austria, a small pilot was performed with almost 35,000 samples, using an MS/MS methodology to quantify enzyme activities. In the pilot screening, two patients with Gaucher’s disease, four with Pompe’s disease and nine with Fabry’s disease were identified. [26]. There is also a small pilot screening study of LSDs in Hungary [29]. They screened about 40,000 samples from the Hungarian newborn screening (NBS) program in Szeged for Fabry, Gaucher, Pompe and Niemann–Pick-A/B disease using MS/MS. After retesting and genetic confirmation three cases of Gaucher, three cases of Fabry, nine cases of Pompe, and two cases with Niemann–Pick were identified, some of which were cases with new mutations and unknown clinical relevance. In Korea, a study was performed including ~35,000 NBS samples from the routine programme that were analysed for alpha-iduronidase (IDUA-)activity using the earlier-described fluorimetric assay [31]. Using a re-test protocol, they requested 19 recall samples identifying after diagnostic two work-up cases of MPS-I. Another Korean-based study investigated the screening for Pompe disease, also demonstrating that identifying infantile Pompe disease early leads to better efficacy of ERT [30,31].

Besides these studies, some countries are currently evaluating LSDs as candidates for routine NBS. Thus, MPS-1 is recommended to be included in the Dutch newborn screening panel, acknowledging that MPS-1 has a severe (Hurler) and a mild (Scheie) phenotype. The Dutch Health council considered two key factors in recommending MPS-I for inclusion in newborn screening. One was the strongly improved efficacy of HSCT (especially through the new possibility of using umbilical cord blood stem cells). The second key factor was the improved performance of screening tests [56]. Screening for Pompe was not recommended, especially on the ground that the available screening method identifies both the infantile as well as the late onset, less severe and less progressive form, which may benefit less from the available ERT therapy.

The UK-NSC recently recommended against adopting MPS-I in the UK newborn screening program [57].

## 9. Conclusions

Screening for several LSDs is now feasible. Methods are available, both commercially and laboratory-developed and -validated, for up to 10 LSDs, sometimes in multiplex assays. A summary of these, as well as incidences, enzymes, biomarkers, and therapeutic options, is given in Table 2. Second-tier methods including biomarker quantification and rapid targeted DNA sequencing to help stratify the positives from first-tier newborn screening are being rapidly advanced. In addition, developments for the addition of new treatment options and the refinement of existing treatment protocols quickly progresses. HSCT is developing into a powerful therapeutic option and, except for MPS-I and Krabbe, may prove effective for other LSDs in the future—for instance, MLD [58].

On the other hand, we are still in the early discovery phase when it comes to screening for some LSDs. A major challenge is the follow up of patients that are predicted to develop late-onset LSDs. We should watch these early newborn screening programs closely so as to best plan additional programs.

## Figures and Tables

**Table 1:** Results of large scale LSD newborn screening pilot studies.

LSD	Number of Screen Positives per 100,000 Newborns			

	WA MS/MS 3-Plex [28]	WA MS/MS 6-Plex [22]	NY MS/MS 2-Plex *	MO DMF-Fluor. 4-Plex [38]
Pompe	16	20	21	48
MPS-I	8	13.6	no data	29
Fabry	15	18	no data	63
Gaucher	no data	6.8	no data	11.4
Krabbe	no data	25	19	no data
Niemann–Pick-A/B	no data	11.4	no data	no data

* (J.Orsini, Wadsworth Center, New York State Department of Health, New York, unpublished results).

Abbreviations: WA; Washington, NY: New York, MO: Missouri.

**Table 2:** Summary of LSDs for which screening methods are available (incidences, enzymes, biomarkers, therapeutic options, screening programmes).

LSD	Incidence (According to Orphanet)	Enzyme	Method of Detection	Biomarkers in Second-Tier Testing or Follow Up Testing	Therapeutic Possibilities	Pilot (P)- or Routine (R) Screening 1
Pompe	1 in 40,000 2	acid alpha-1,4-glucosidase	MS/MS, fluorimetry	glucose tetramer, creatine kinase	ERT	R: Taiwan, MO, NY, OH, KY, IL P: Austria 3, NJ, WA
Hurler/Scheie (MPS-1)	1–9 in 1,000,000	alpha-L-iduronidase	MS/MS, fluorimetry	heparan sulphate, dermatan sulphate 4	ERT, HSCT	R; Taiwan, MO, NY, KY, IL P: NJ, WA, PA
Fabry	1–5 in 10,000	alpha-galactosidase A	MS/MS, fluorimetry	lyso-Gb3, tissue Gb3	ERT	R: MO, IL, Taiwan P: NJ, WA
Gaucher	1–9 in 100,000	beta-glucosidase	MS/MS, fluorimetry	Glucosylsphingosine	ERT, SRT	R: MO, IL, Taiwan P: NJ, WA, Austria
Krabbe	1–9 in 100,000	galactosylceramidase	MS/MS, fluorimetry	Psychosine (galactosyl-sphingosine)	HSCT	R: MO, NY, KY, OH P: WA, NJ
Niemann–Pick A/B	<1 in 1,000,000 (A), 1–9 in 1,000,000 (B)	sphingomyelin phosphodiesterase-1	MS/MS, fluorimetry	lysosphingomyelin	SRT	R: IL P: WA, Austria,
Metachromatic leukodystrophy	1–9 in 1,000,000	arylsulfatase A	MS/MS	-	HSCT	P: WA

1 MPS-II, MPS-IVA, MPS-VI, MPS-VII are also pilot screening programmes in Washington and are included in routine screening in Taiwan (except for MPS-VII);

2 Incidence as cited in OMIM;

3 the Austria pilot programme has been put on hold;

4 may be a biomarker for other LSDs as well.
